# Acquired Myasthenia Gravis in a Juvenile Australian Shepherd Dog and Efficacy of Leflunomide Treatment

**DOI:** 10.1155/crve/1607891

**Published:** 2025-08-13

**Authors:** Alejandra Mondino, Gilad Fefer, Anna Tauro, Natasha J. Olby, Melissa J. Lewis, Christopher L. Mariani

**Affiliations:** Department of Clinical Sciences, North Carolina State University, Raleigh, North Carolina, USA

## Abstract

Myasthenia gravis is a disorder of neuromuscular transmission characterized by muscle weakness and exercise intolerance that is rarely diagnosed in dogs younger than 6 months. We report a case of juvenile-onset myasthenia gravis in a 5-month-old, intact male Australian Shepherd with onset of clinical signs at 4 months of age. The dog presented with a 4-week history of progressive weakness and regurgitation. Diagnosis was confirmed by an elevated serum acetylcholine receptor antibody concentration. Treatment with pyridostigmine provided only partial improvement. Leflunomide was added to achieve immunosuppression while avoiding the side effects of corticosteroids, such as muscle weakness. This treatment combination was well-tolerated; the dog went into clinical remission, including resolution of megaesophagus, and progressive reduction in acetylcholine receptor antibody concentration was noted. This report highlights the importance of considering myasthenia gravis in juvenile dogs and emphasizes the utility of measuring acetylcholine receptor antibodies in suspected cases regardless of age. The safe and possibly efficacious use of leflunomide in a dog with this disease is also demonstrated. Further studies are needed to characterize the juvenile presentation of this disease and to evaluate the efficacy of treatment with leflunomide as the sole immunosuppressant medication in a larger canine population.

## 1. Introduction

Myasthenia gravis (MG) is a well-characterized disorder of neuromuscular transmission marked by skeletal muscle weakness and fatigability [1, 2]. MG is associated with autoantibodies against nicotinic acetylcholine (ACh) receptors at the neuromuscular junction. Congenital myasthenic syndromes (CMSs) are inherited disorders characterized by clinical signs similar to MG but occur in the absence of autoantibodies. These syndromes can be classified based on the location of the defect within the neuromuscular junction. Presynaptic forms affect ACh synthesis, such as the mutation in the choline acetyltransferase (CHAT) gene described in Danish Pointing Dogs [3]. Synaptic forms result from acetylcholinesterase (AChE) deficiency, as seen with mutations in the COLQ gene, which encodes the collagenous tail subunit of AChE, reported in Golden Retrievers [4]. Postsynaptic forms include mutations in the CHRNE gene, which encodes the epsilon subunit of the ACh receptor, and have been identified in Jack Russell Terriers [5], Smooth Fox Terriers [6], and English Springer Spaniels [7].

Age of onset of MG in dogs has a bimodal distribution, with peaks at 3 and 10 years of age [8]. The mean age of onset is approximately 6 years [9]. Dogs younger than 1 year of age are less likely to present with the disease [10] with only rare cases reported in dogs younger than 6 months [11, 12]. In contrast, dogs with CMS typically exhibit clinical signs between 6 and 12 weeks of age [1, 12, 13].

A cornerstone treatment for MG is symptomatic therapy with AChE inhibitors, such as pyridostigmine, which prolong the presence of ACh in the synaptic cleft, thereby improving neuromuscular transmission [13, 14]. While spontaneous remission can occur in dogs, AChE inhibitors do not address the autoimmune pathogenesis of the disease [15]. Prednisone is commonly used as a first-line immunosuppressant for various immune-mediated disorders, including MG, due to affordability and rapid onset of action [14, 16, 17]. However, corticosteroids have a multitude of side effects, including exacerbation of neuromuscular weakness, which can complicate management of the disease [18]. Several more targeted immunosuppressive therapies have been described as treatments for MG in dogs, including azathioprine, cyclosporine, and mycophenolate mofetil [19–22]. Leflunomide, an inhibitor of T and B cell proliferation, has been shown to be effective in experimental rodent models of MG [23] and in people with MG [24, 25] but has not been described for the treatment of MG in dogs.

Here, we report a case of juvenile-onset MG in an Australian Shepherd dog managed successfully with pyridostigmine and leflunomide.

## 2. Case Presentation

A 5-month-old, 10.6 kg, intact male Australian Shepherd was presented to the North Carolina State University (NCSU) Veterinary Hospital's Emergency Service with a history of progressive weakness, intermittent difficulty walking, and regurgitation that started when the dog was 4 months old. The owners initially observed intermittent paraparesis, as well as difficulty jumping and climbing stairs. The dog was seen by his primary veterinarian, and physical and orthopedic examinations were found to be normal. A complete blood cell count was within normal limits. A biochemistry panel showed mild hyperphosphatemia (8.1 mg/dL, reference interval: 2.5–6.1), which was considered normal for the dog's age. Serum total T4 was within the reference interval. Although radiographs of the lumbar spine, pelvis, and proximal pelvic limbs were unremarkable, the dog was treated with a nonsteroidal anti-inflammatory drug (carprofen, dose unknown) and gabapentin (dose unknown) for suspected panosteitis. After a lack of response to this treatment, a tapering course of prednisone was prescribed for suspected T3-L3 myelopathy (0.47 mg/kg q 12 h for 4 days, q 24 h for 4 days, and then q 48 h for 2 weeks), leading to a 75% improvement in clinical signs according to the owners. Upon reducing the prednisone from daily to every other day, the dog's clinical signs worsened, with the development of tetraparesis and occasional regurgitation.

On initial presentation to NCSU, general physical examination was unremarkable. Neurological examination revealed ambulatory tetraparesis characterized by a short-strided gait in all limbs, which progressed to nonambulatory status following mild exercise. The dog was able to regain ambulation after rest. Postural reactions, cranial nerve examination, and segmental spinal reflexes were within normal limits. Regurgitation was not noted during hospitalization. The dog's clinical signs were localized to the neuromuscular system with a disorder of the neuromuscular transmission or myopathy suspected. Neostigmine administration (0.04 mg/kg IM) yielded a positive response within 10 min (Supporting Information 1: Video S1). Thirty minutes after completion of this test, electrodiagnostic evaluation was performed under general anesthesia including electromyography (EMG), motor nerve conduction velocity, F-wave testing, and repetitive nerve stimulation. The EMG showed rare fibrillation potentials in the palmar and plantar interosseous muscles but was unremarkable in all other muscles. Motor nerve conduction velocities of the left sciatic-tibial and left ulnar nerves were 58 and 51 m/s, respectively (reference interval [for dogs between 3 and 6 months]: 53 ± 2.8 and 54.1 ± 2.3) [26]. F-waves following left tibial nerve stimulation were within normal limits with an *F*-ratio of 1.93 (reference interval: 1.95 ± 0.086) [27]. Repetitive nerve stimulation of the tibial nerve (recorded from the interosseous muscle) was unremarkable, with no decrement observed at 5, 10, or 20 Hz.

Biopsies of the right cranial tibial and triceps brachii muscles were performed and submitted to the Comparative Neuromuscular Laboratory at the University of California, San Diego. Samples were submitted both as cold, fresh tissue (placed in a dry, watertight container and refrigerated until shipment on cold packs) and as fixed tissue (immersed in 10% neutral buffered formalin). Complete muscle profiles on both muscle samples [28] showed no abnormalities. ELISA antibody testing (IgG and IgM) for *Toxoplasma gondii* and indirect fluorescence antibody testing for *Neospora caninum* on serum were negative. Serum AChR antibody concentration was 2.56 nmol/L (reference interval: < 0.6 nmol/L), consistent with acquired MG.

Pyridostigmine was initiated at 0.5 mg/kg PO q 12 h, resulting in transient improvement of clinical signs. After a few weeks, weakness recurred, potentially due in part to continued growth of the dog resulting in a lower dosage (0.44 mg/kg). The pyridostigmine dose was gradually increased (up to 1.25 mg/kg PO every 8 h), resulting in only transient improvement with progressively shorter duration of benefit (less than a week). One month after the initial diagnosis, the dog represented at the NCSU Emergency Service for marked worsening of tetraparesis and much more frequent regurgitation. Repeat AChR antibody concentration showed an increase to 7.16 nmol/L. Thoracic radiographs showed megaesophagus with no evidence of pneumonia or other abnormalities. Immunosuppressive therapy with leflunomide (3.3 mg/kg PO q 24 h) was initiated, and pyridostigmine was further increased to 1.45 mg/kg PO every 8 h. In addition, the owners were instructed to feed the dog small meatballs from an elevated food bowl.

A recheck evaluation conducted 3 weeks after this change in treatment showed marked clinical improvement (Supporting Information 2: Video S2) and resolution of regurgitation. AChR antibody concentration had decreased to 2.3 nmol/L at this time. Due to increased frequency of urination and improvement in clinical signs, the pyridostigmine dose was reduced to 1 mg/kg PO every 8 h for 2 weeks and then to 1 mg/kg every 12 h. Urination normalized, and the dog remained clinically normal. The pyridostigmine was gradually tapered until discontinuing it 4 months after diagnosis. Leflunomide was continued at the same dosage.

Six months postdiagnosis, the dog returned to NCSU for a recheck evaluation. Physical and neurological examinations were unremarkable, and thoracic radiographs showed resolution of the megaesophagus. AChR antibody concentration had decreased to 1.76 nmol/L. At the time of writing, 11 months after diagnosis, the dog remains asymptomatic on leflunomide, with an AChR antibody concentration of 1.32 nmol/L.

## 3. Discussion

Here, we report the diagnosis of MG in a puppy with an onset of clinical signs at 4 months of age and successful treatment using a combination of leflunomide and pyridostigmine. Initially, the dog was treated with pyridostigmine alone, which produced transient improvement but became progressively less effective despite multiple dose increases. At higher doses, the dog also demonstrated increased urination, a potential side effect of pyridostigmine [29]. When leflunomide was added, the dog achieved resolution of clinical signs within a few weeks, with no relapses, even as pyridostigmine was tapered and eventually discontinued. Additionally, while esophageal muscles often respond poorly to anticholinesterase therapy [30], this dog's megaesophagus resolved. Although the AChR antibody concentration in this case had continued to decline, at the time of writing, it remained above the reference range. This raises the possibility that immunologic remission may lag behind clinical improvement. In a retrospective study comparing pyridostigmine alone to pyridostigmine combined with mycophenolate mofetil, the time to remission was numerically longer in the mycophenolate group, although the difference was not statistically significant [21]. Continued monitoring of antibody titers in future cases may help determine whether serologic and clinical remission occur in parallel or at different rates.

MG is a well-known neuromuscular disease in dogs. The acquired form of MG can affect dogs of any age but typically exhibits a bimodal age distribution, with peaks at 3 and 10 years old [8]. In a large case series of more than 1000 dogs with MG, dogs aged 1–15 years and those older than 16 years were more likely to develop MG compared to dogs younger than 1 year (odds ratio: 1.4 and 3.6, respectively) [10]. Another study of 94 dogs with MG reported an age range of 8 months to 14 years, with no dogs younger than 6 months [9]. Rare reports of juvenile-onset MG in dogs have documented individuals as young as 7 weeks [12], 3 months [11], and 8 months [9]. While uncommon, this case report reinforces that MG can occasionally manifest in dogs younger than 6 months and highlights the importance of evaluating serum AChR antibody concentration, regardless of the dog's age.

Due to its very low prevalence in juvenile cases, little is known about the clinical presentation of MG in this age group. In this case, the clinical signs were typical of generalized MG, including generalized weakness, exercise intolerance, and regurgitation with megaesophagus [8, 9]. Although the generalized weakness is similar to other reports of juvenile dogs with MG [9, 11], regurgitation and megaesophagus were not reported in prior cases [11, 12]. Clinical presentation in dogs with CMS overlaps with MG, although megaesophagus is very rare and has only been described in the Smooth terrier breed [6]. Given the rarity, it remains unclear if the risk of megaesophagus in juvenile MG is lower.

The diagnosis of MG was confirmed by the detection of a high AChR antibody concentration, the gold-standard test for MG in dogs. In addition, the dog showed a positive response to neostigmine administration. While this is suggestive of MG, false positives can occur, as in some cases of polymyositis [31]. EMG showed no abnormal spontaneous activity except for rare fibrillation potentials in the interosseous muscles. Motor nerve conduction velocity and F-waves were unremarkable. These results, together with normal muscle biopsy findings, excluded other possibilities and further supported a diagnosis of MG. Unexpectedly, the repetitive nerve stimulation study was normal. When testing at a low stimulation frequency (< 5 Hz, typically 2–3 Hz), a decrement of > 10% in the amplitude of recurrent compound muscle action potentials is expected with MG [27, 32]. In this dog, due to an issue with the software employed, stimulation frequency less than 5 Hz was not possible. In addition, electrodiagnostic testing was performed approximately 30 min after the neostigmine test, which could have affected the results [33].

The use of immunosuppressant drugs in dogs with MG remains controversial due to the high risk of aspiration pneumonia and the potential for achieving disease remission without immunosuppression [15]. In this case, the response to pyridostigmine alone was transient and incomplete, and immunosuppression helped to achieve a remission of clinical signs. Corticosteroids, especially prednisone, are commonly used to treat immune-mediated diseases in dogs [34], and this dog initially showed improvement with an anti-inflammatory dose of prednisone prescribed by the primary veterinarian. However, we opted not to use corticosteroids in this case due to their potential to worsen neuromuscular weakness and cause side effects such as polyuria, polydipsia, polyphagia, gastric ulceration, restlessness, and excessive panting, particularly at the higher doses required for immunosuppression [17, 34, 35]. Moreover, corticosteroids inhibit the function of a variety of immune cells, which can be particularly hazardous for patients at risk for aspiration pneumonia [36].

Alternative immunosuppressive treatments that specifically target lymphocytes and offer fewer side effects have been suggested for managing MG in dogs. Azathioprine is commonly used in people with MG [37] and has been reported in dogs with MG [17, 19]. However, improvement after drug initiation can be delayed for 2–8 weeks, making it less ideal when a more rapid response is needed. Cyclosporine offers the advantage of a quick therapeutic effect [22], although higher cost can be a limiting factor. Additionally, pharmacokinetic studies have shown that blood concentrations are highly variable between individuals, and therefore, serum drug concentration monitoring should be considered in these dogs [38–40]. Mycophenolate mofetil has also been identified as a potential treatment option for MG. Although case reports have demonstrated its potential effectiveness [20, 41], a retrospective study comparing dogs treated with both mycophenolate and pyridostigmine to those treated with pyridostigmine alone did not reveal an additional therapeutic benefit from mycophenolate [21].

Leflunomide is another immunosuppressant described for the treatment of MG in humans. It is an isoxazole derivative that exerts its immunomodulatory effects via its main metabolite, A77 1726 or teriflunomide, which inhibits the proliferation of T and B cells by inhibiting dihydroorotate synthase, an enzyme critical for pyrimidine biosynthesis [42]. Leflunomide has been demonstrated to be effective and well tolerated in people with MG, helping to reduce the dosage of steroids required for these patients [24, 25]. Moreover, in rodents with experimentally induced MG, it can ameliorate the disease severity by regulating humoral immune responses, inhibiting Th1 cells, and promoting Treg cells [23, 43]. Leflunomide has been described as a treatment option in other immune-mediated diseases in dogs [44, 45] and is usually well tolerated, with gastrointestinal upset being the main reported side effect. Leflunomide also has the advantage of requiring only once-daily administration [46].

In conclusion, while rare, acquired MG can present in juvenile dogs. Serum AChR antibody concentration should be measured in suspected MG cases regardless of the patient's age. Additionally, leflunomide in combination with pyridostigmine might represent an effective and safe treatment option for dogs with acquired MG. Future studies could evaluate this treatment regimen in a group of myasthenic dogs and compare its efficacy with other immunosuppressive medications.

## Data Availability

The data that support the findings of this study are available from the corresponding author upon reasonable request.

## Supporting Information

Supporting Information 1 Video S1. Neostigmine test. This video demonstrates the neostigmine test. Before administration, tetraparesis is evident, characterized by a short-strided gait in all limbs, which progresses to non-ambulatory status after mild exercise. A positive response, with clear improvement in gait, is observed 10 min after neostigmine administration.

Supporting Information 2 Video S2. Recheck appointment. This video shows marked improvement 3 weeks after initiating leflunomide treatment.
